# Supplementary material for: High PD-L1 Expression Correlates with an Immunosuppressive Tumour Immune Microenvironment and Worse Prognosis in ALK-Rearranged Non-Small Cell Lung Cancer
Source: Biomolecules. 2023 Jun 15;13(6):991. doi: 10.3390/biom13060991 (PMC10296689; doi:10.3390/biom13060991)
Supplement: Supplementary file 1 [file biomolecules-13-00991-s001.zip › Table S1.pdf]

Table S1. Clinical outcomes of 16 patients with ALK-rearranged NSCLC treated with crizotinib.

PD-L1hi group (n=7)						PD-L1lo group (n=9)					
case	PD-L1	Treg	CD8+PD-1+	PFS	OS	case	PD-L1	Treg	CD8+PD-1+	PFS	OS
1	50%	high	low	2.5	15.8	1	0%	low	low	47.6	47.6
2	50%	high	low	9.8	20.2	2	0%	low	high	21.7	49.8
3	65%	high	high	2.8	24.0	3	0%	low	low	44.9	52.0
4	65%	low	low	15.0	17.5	4	0%	low	high	26.4	51.7
5	70%	high	high	3.2	31	5	0%	high	low	51.1	63.0
6	80%	low	high	2.9	**51.4**	6	0%	low	high	35.5	63.7
7	90%	high	low	10.7	15.1	7	2%	high	low	8.5	45.7
						8	5%	low	low	8.5	55.0
						9	10%	low	low	33.5	36.3
