# Supplementary figures and images for: High PD-L1 Expression Correlates with an Immunosuppressive Tumour Immune Microenvironment and Worse Prognosis in ALK-Rearranged Non-Small Cell Lung Cancer
Source: Biomolecules. 2023 Jun 15;13(6):991. doi: 10.3390/biom13060991 (PMC10296689; doi:10.3390/biom13060991)

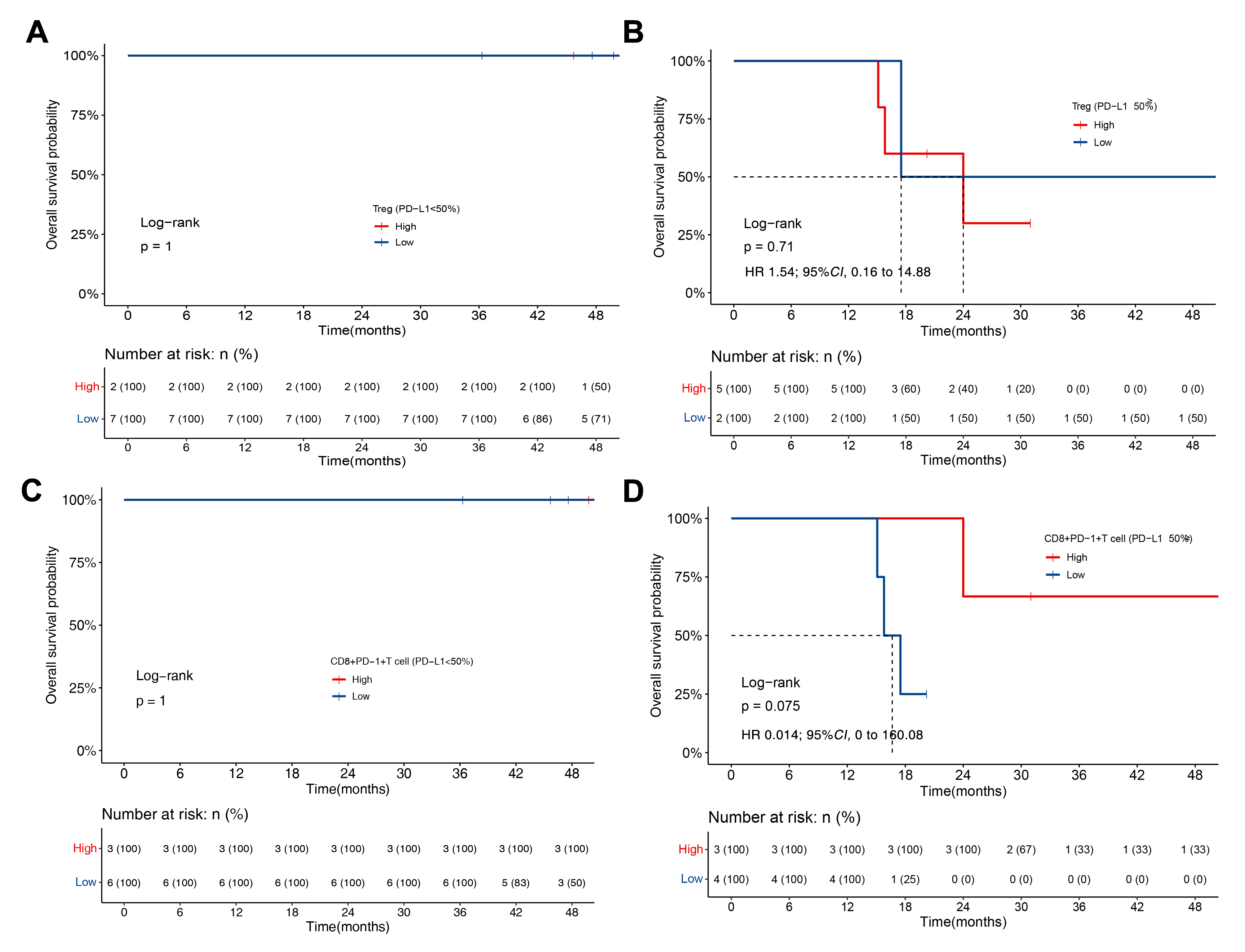

Supplement: Supplementary file 1 [file biomolecules-13-00991-s001.zip › Figure S1.tif]
